# A key to unlocking the door: water pressure method for endoscopic submucosal dissection of a superficial non-ampullary duodenal epithelial tumor with a history of multiple biopsies

**DOI:** 10.1055/a-2218-2670

**Published:** 2024-01-09

**Authors:** Tao Dong, Lin Jing, Yaohui Wang, Jun Xiao

**Affiliations:** 1 375808 Digestive Endoscopy Center, Affiliated Hospital of Nanjing University of Chinese Medicine, Nanjing, China; 2 375808 Department of Pathology, Affiliated Hospital of Nanjing University of Chinese Medicine, Nanjing, China

Endoscopic resection of superficial non-ampullary duodenal epithelial tumors has been popular for its mini-invasiveness and comparable treatment outcome to surgery. However, the thin wall and narrow submucosal space of the duodenum make endoscopic treatment difficult 1 . Furthermore, preoperative biopsy remains common and could induce significant submucosal fibrosis. The underwater method has been reported to facilitate the resection, but sometimes a sufficient amount of saline cannot be maintained to soak the lesion 2 3 . In this case, we emphasized the role of the water pressure method in assisting endoscopic submucosal dissection (ESD) for a superficial non-ampullary duodenal epithelial tumor with a history of multiple biopsies ( Video 1 ).

Water pressure method facilitating endoscopic submucosal dissection of a superficial non-ampullary duodenal epithelial tumor.Video 1

A 52-year-old man was referred for endoscopic treatment of a superficial non-ampullary duodenal epithelial tumor in the descending duodenum ( Fig. 1 ). Notably, the tumor had undergone biopsies at the prior two esophagogastroduodenoscopy examinations, of which histology revealed low grade tubular adenoma. Given the potential of submucosal fibrosis, ESD was scheduled with a curative intent. After circumferential mucosa incision, submucosal dissection proceeded but became difficult due to poor visualization of the submucosal layer at the lateral edge ( Fig. 2 ). Due to the non-lifting sign, the water pressure method was applied to assist. Normal saline was irrigated via waterjet to hit the submucosa, which effectively lifted the collapsed mucosal flap, facilitating identification of dissection line ( Fig. 3 ). The operation was accomplished smoothly with a clear vision with the assistance of the water pressure method. The lesion was resected en bloc ( Fig. 4 **,** Fig. 5 ) and the defect was closed with several clips. No intraoperative complication occurred and the postoperative course was uneventful. Histopathology demonstrated tubular adenoma with a focal high grade intraepithelial neoplasia and negative margins.

**Fig. 1:**
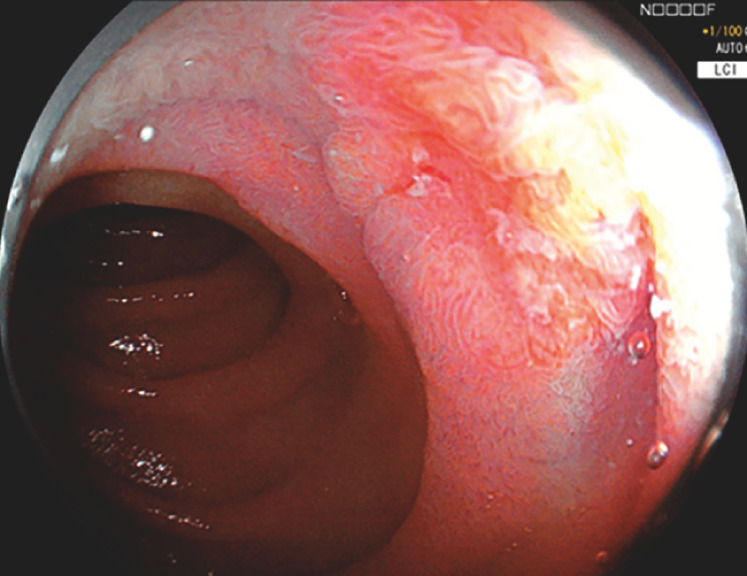
Linked color imaging showed a 15 × 12-mm 0-IIa+IIc orange-reddish lesion at the descending duodenum.

**Fig. 2:**
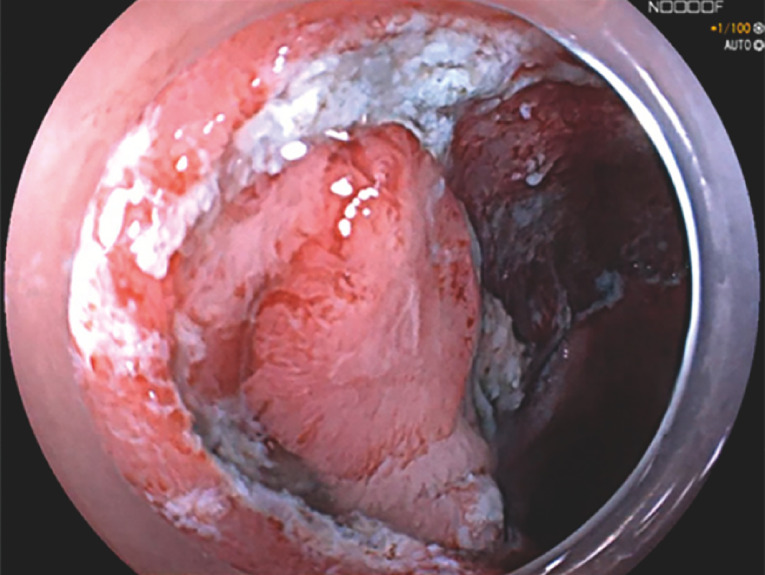
The mucosal flap remained collapsed after multiple sessions of submucosal injection.

**Fig. 3:**
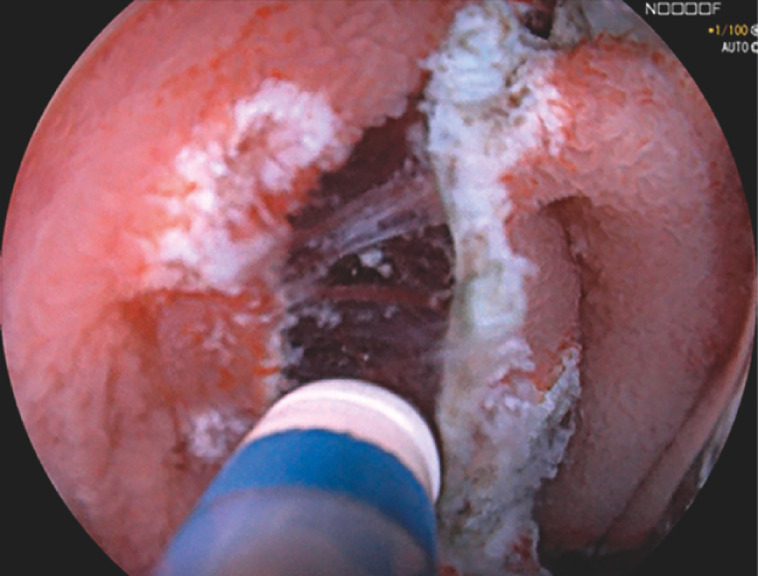
With the water pressure method, the mucosal flap was effectively lifted by the active water stream, facilitating the visualization of the submucosal fibers.

**Fig. 4:**
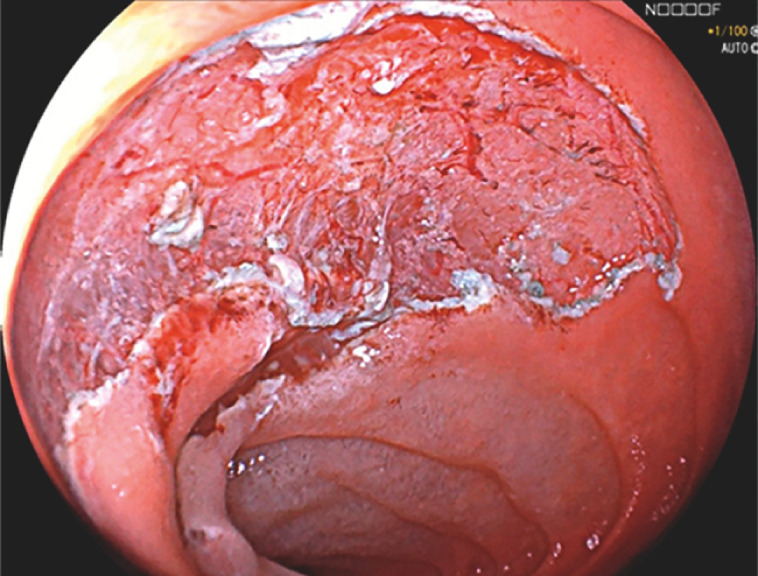
The defect after complete resection without muscular injury.

**Fig. 5:**
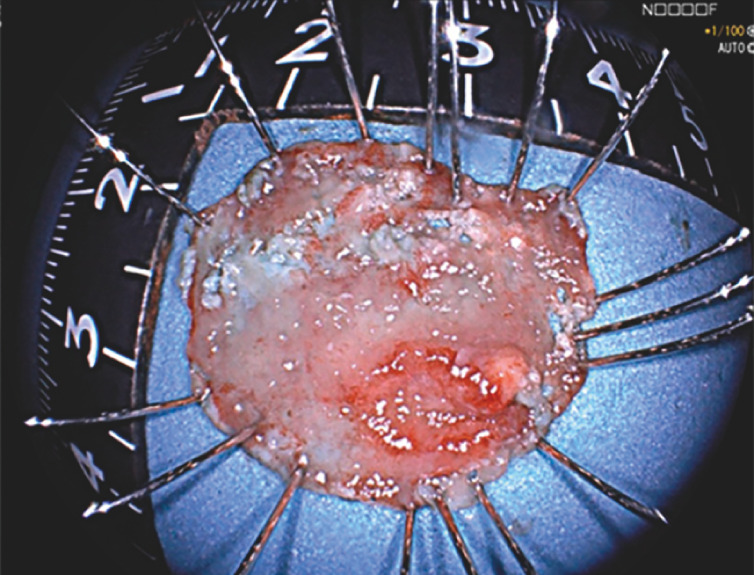
The resected specimen.

Compared to the underwater method, the water pressure method additionally provides an active water stream that acts as a key to open the submucosal space for endoscopists and hence should be considered as a useful adjunct in the case of superficial non-ampullary duodenal epithelial tumors with a history of biopsy.

Endoscopy_UCTN_Code_TTT_1AO_2AG

## Notes

Endoscopy E-Videos https://eref.thieme.de/e-videos
*E-Videos* is an open access online section of the journal *Endoscopy* , reporting on interesting cases and new techniques in gastroenterological endoscopy. All papers include a high-quality video and are published with a Creative Commons CC-BY license. Endoscopy E-Videos qualify for HINARI discounts and waivers and eligibility is automatically checked during the submission process. We grant 100% waivers to articles whose corresponding authors are based in Group A countries and 50% waivers to those who are based in Group B countries as classified by Research4Life (see: https://www.research4life.org/access/eligibility/ ). This section has its own submission website at https://mc.manuscriptcentral.com/e-videos .
